# Transparent Display using a quasi-array of Si-SiO_2_ Core-Shell Nanoparticles

**DOI:** 10.1038/s41598-019-38771-9

**Published:** 2019-02-19

**Authors:** Mahboubeh Dolatyari, Ali Jafari, Ali Rostami, Axel Klein

**Affiliations:** 1 SP-EPT Lab., ASEPE Company, Industrial Park of Advanced Technologies, Tabriz, 5364196795 Iran; 2 OIC Research Group, Faculty of Electrical and Computer Engineering, University of Tabriz, Tabriz, 5166614761 Iran; 3 Department of Chemistry, Institute for Inorganic Chemistry, University of Cologne, Greinstrasse 6, D-50939 Cologne, Germany

## Abstract

A novel type of transparent monitor with high-resolution images based on Si-SiO_2_ core-shell nanoparticles is presented in this contribution. In this monitor, a quasi-array of nanoparticles was used to obtain a very sharp scattering profile. For this purpose, the Si-SiO_2_ nanoparticles were synthesized and with controlling the size of particles, the dominant emission wavelength was controlled. For the fabrication of a blue color transparent monitor the solution processed Si-SiO_2_ nanoparticles were dispersed in polystyrene and then coated on a transparent glass surface. After drying the film, the typical features representing a transparent monitor were studied. A video projector was used and text and pictures were sent on the monitor. This monitor reveals very attractive features such as simplicity, wide viewing angle, scalability to larger sizes and low cost. Importantly, the texts and pictures can be well presented on both sides of the fabricated monitor. The composite thin film can be also separated from the glass and can be used as a flexible display. To shed light on the impact of the structure on the optical properties Si-SiO_2_ and Ag nanomaterials representing perfect arrays of nanoparticles, quasi-arrays and randomly oriented nanoparticles were calculated/simulated using the finite-difference time-domain (FDTD) method. The results were compared to the experimental data and show a high accordance.

## Introduction

Transparent displays are ‘see-through’ screens: a person can simultaneously view both the graphics on the screen and real-world content. The ability to display photos and texts on a transparent display would have attractive and useful applications. Here are some applications of transparent displays: displaying driver related information on the windscreen to enhance the driver’s precision and comfort^1,2^, displaying advertisement on shop front windows or displaying information on eye glasses including entertainment purposes. Transparent displays fall into two general categories: those that emit light, and those whose light is provided by a projector^3^. Displays that emit light such as OLEDs and LCDs are prevalent today^4–6^. Among displays for which the image is provided by a projector one of the simplest approaches is known as “head-up” display^7^. The structure of this kind of display consists of two mirrors that guide through the light from the main display to the viewer’s eye. This display is suitable for defined situations. However, the main problem with the display is a narrow viewing angle which affects the viewer’s position. Diffusive displays have solved the viewing angle problem by scattering the projector’s light^8,9^. An immediate problem concerning this type of setup is that most of the light from the projector passes through the transparent panel instead of being scattered resulting in low image brightness. In order to solve this problem, researchers have used different materials to enhance transparency while maintaining the high resolution of the image. The fluorescent material converts ultraviolet or infrared light into visible light^10–13^. Displays made from these materials have high transparency but their efficiency is usually low. A different approach for displays is the use of spherical silver nanoparticles^14^ which selectively scatter blue wavelengths and provide lower emission for other wavelengths. Due to the broad scattering the transparency of the display is slightly reduced. Since the display is a monochrome display, a much narrower emission is needed to create a full-color display. These limitations motivated us to find an innovative way to create a transparent display. In this paper, we report on the synthesis and quantum-chemical simulation of an optimized quasi-array^15^ containing spherical silicon/silicon oxide core/shell nanoparticles to create a blue-colored transparent display. The fabrication method used in this work is the solution processed method known as “Doctor Blade Method” which is a simple method and applicable to large-scale production. In contrast to other reported methods like^16^ which uses normal planar technology this method is not expensive. The resolution in the planar technology depends on lithography which is an expensive procedure and large size displays cannot be fabricated.

## Mathematical Method and Theoretical Background

The optical wave emission from the proposed transparent display can be evaluated by solving Maxwell’s equations^17,18^. To extract the absorption and emission coefficients for the structure, Maxwell’s equations must be solved by using a boundary condition that is the incident light in this case. In this work, array like systems of core-shell silicon-silicon dioxide nanoparticles uniformly dispersed in polystyrene were coated on transparent silica glass and we consider the crystalline-like core-shell nanoparticles in the polystyrene host as uniformly distributed. The periodicity of the structure is controlled by the nanoparticle’s concentration. Absorption, emission and extinction coefficients were calculated using the finite-difference time-domain (FDTD) method (see below) and compared with experimental data to evaluate the performance of the structure. One of the best quantities to evaluate these coefficients is the so-called cross-section^18^. The cross-section quantity is defined as the net rate at which electromagnetic energy (W) crosses the surface of an imaginary sphere of radius r ≥ R centered on the particle divided by the incident irradiance (I_i_). Thus, the net rate (W) at which electromagnetic energy crosses the boundary of a closed surface (A) which encloses a volume (V) and it is:

1 $$W=-\,{\int }_{A}(E\times H).\mathop{n}\limits^{\frown {}}dA$$

where E, H, and n are the electric field, magnetic field and the normal vector to area A.

To quantify the rate of the absorbed (W_abs_) or emitted (W_sca_) electromagnetic energy by the transparent display, the absorption (C_abs_) or emission (C_sca_) cross-sections are defined in equations (2) and (3).

2 $${C}_{abs}=\frac{{W}_{abs}}{{I}_{i}}$$

3 $${C}_{sca}=\frac{{W}_{sca}}{{I}_{i}}$$

In the same way, the extinction cross-section can be defined as:

4 $${C}_{ext}={C}_{sca}+{C}_{abs}$$

The absorption and emission cross sections allow to quantify the amount of energy removed from the incident field due to emission and/or absorption made by the core-shell nanoparticles. By dividing these cross sections to the geometrical cross-section area of the particle projected onto a plane perpendicular to the incident beam (G), we obtain the emission, extinction and absorption efficiencies. For spherical nanoparticles G = π.r^2^ and the efficiencies are expressed as in equations (5) and (6).

5 $${{\sigma }}_{sca}=\frac{{C}_{sca}}{G}$$

6 $${{\sigma }}_{abs}=\frac{{C}_{abs}}{G}$$

In the past few years, a number of numerical methods have been developed for describing nanoparticle arrays and aggregates with complex structures including the discrete dipole approximation (DDA)^19,20^, the multiple multipoles (MMP)^21^, the finite-difference time-domain (FDTD)^22^, and the T-matrix method^23,24^. The finite-difference time-domain (FDTD) method is a technique used for directly solving Maxwell’s equations in the time domain. FDTD discretize the entire volume of the transparent display to numerous Yee cells and then solve Maxwell’s equations as a function of time numerically. We used this method repeatedly to simulate the emission and absorption cross-section of the proposed structures in order to find the most optimal structure.

## Results and Discussion

### Calculation/Simulations

**Figure 1:**
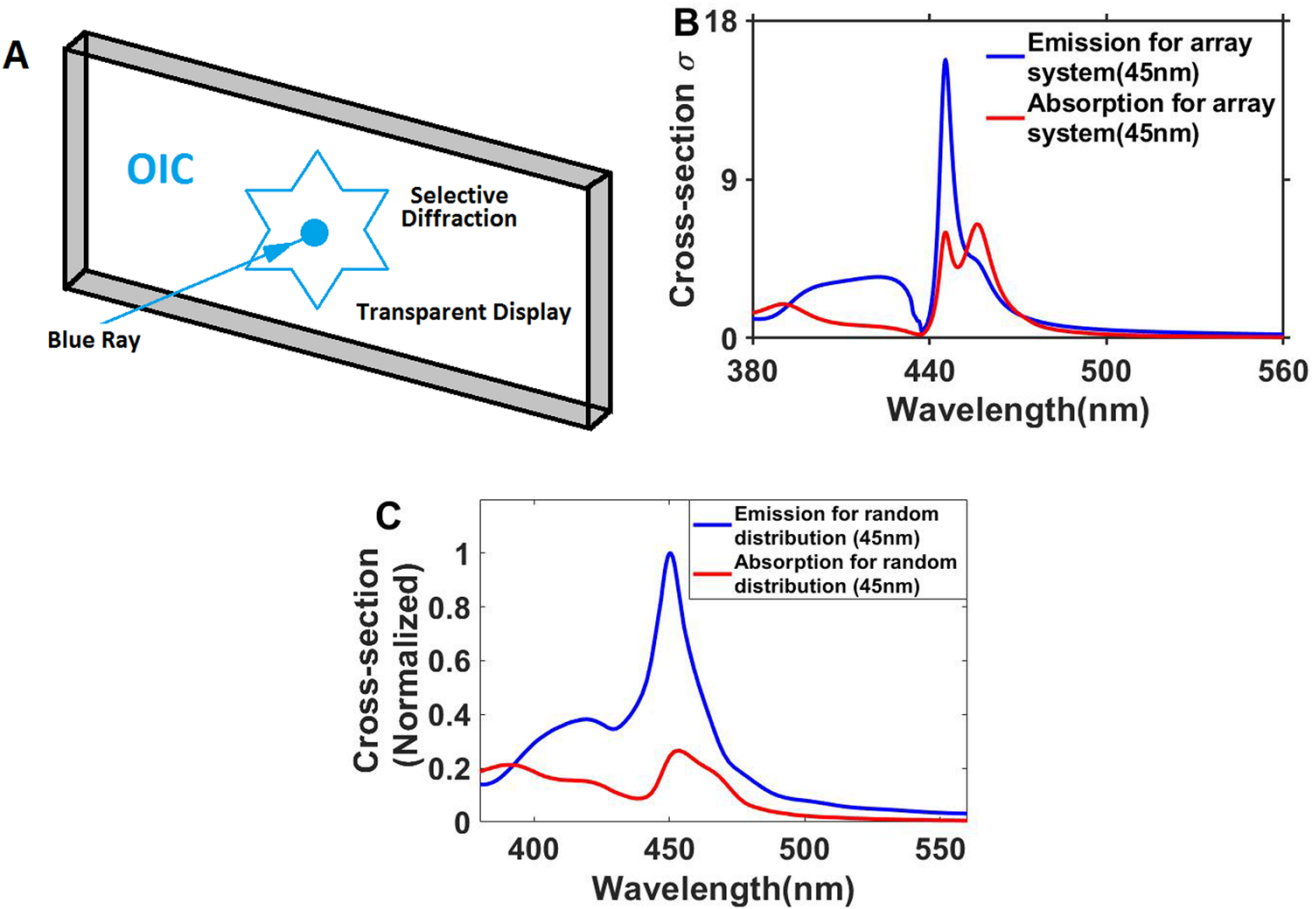
(**A**) Schematic description of a transparent display, (**B**) Calculated cross-section profiles for Si-SiO_2_ nanoparticles in a periodic structure (nanoparticle radius is 45 nm, the period is 301.7 nm) and (**C**) Calculated cross-section profiles for Si-SiO_2_ nanoparticles with random distribution of particles with radius between 40 and 50 nm (Paliks data^37^ is used for optical constants).

In composite materials^25^ as the nanoparticles inside the polystyrene host in the here presented material, non-uniform particles and random distribution of nanoparticles lead to a broad distribution profile and sharp peaks in the frequency domain normally cannot be obtained^26–32^. This is the main reason for overlapping of emissions at different wavelengths resulting in interference and high-resolution images can thus not be obtained. In the here presented structure for a transparent display we used an array of nanoparticles close to the periodic case to allow very sharp peaks^33–36^. In this situation overlaps of different emissions e.g. between red, green and blue colors will be low and high contrast images can be expected. To make an array of nanoparticles, we synthesized uniform Si-SiO_2_ core-shell nanoparticles, dispersed them thoroughly in polystyrene (PS) and then spin-coated the mixture on a transparent glass surface. By embedding such an array of nanoparticles in a transparent medium and by projecting images at the resonant wavelength, we can create a screen that scatters most of the projected light while being almost transparent to the broadband ambient light (Fig. 1(A)). The screen should thus be transparent except for the light at resonance wavelength.

Figure 1(B) show the calculated cross-section for the Si-SiO_2_@PS array with the spheres having approx. 45 nm radii, and a period of 301.7 nm, using the finite-difference time-domain (FDTD)^22^ method and Paliks^37^ model for the permittivity. The absorption cross-section is negligible in this case.

Figure 1 illustrates nicely that wavelength selectivity is achievable for an array of spherical Si-SiO_2_ nanoparticles. Generally, the resonance frequencies and spectral resolutions found for structures of periodic nanoparticles strongly depend on the size, the shape of nanoparticles, periodicity, and the dielectric environment of nanoparticles^38–41^. For a rough estimate to determine which structures are optimum to use^14^ a figure of merit (FOM) can be defined as follows.

7 $$FOM=\frac{{{\sigma }}_{sca}({\lambda }_{0})}{2{\bar{{\sigma }}}_{sca}+\,\max \,\{{{\sigma }}_{abs}\}}$$

Equation (7), shows that the FOM is maximum for minimum absorption cross-section (in the range 390 nm to 750 nm), maximum for high scattering cross section in the desired wavelength and low in other wavelengths. On the other hand, introducing a very sharp scattering profile is critical in transparent monitor design. The numeral 2 used in the denominator is a weighing factor chosen to balance sharp scattering and low absorption. The absolute value of the cross-section per period of the periodic structure is less important here because one can adjust the 2-D density of the nanoparticles on the screen; thus the FOM is defined as a ratio. Also, in order to have a colorless transparent screen, we prefer a flat absorption spectrum. So we use max {*σ*_*abs*_} rather than ${\bar{{\sigma }}}_{abs}$. One can also consider imposing a wavelength-dependent weight on the cross-sections to account for the spectral sensitivity of human eyes, but we omit this weight for simplicity. With this definition for FOM, we performed numerical optimizations on the proposed structures of spherical silicon nanoparticles, embedded in a transparent medium of silica. Emission and absorption cross-sections are calculated with the FDTD method, using experimental values of the dielectric function for silica and silicon. Data for the cross-section of structures optimized to scatter monochromatic light at 445 nm (blue), 526 nm (green), and 636 nm (red) and the corresponding particle sizes and FOMs are listed in Table 1.

**Table 1:** Optimal information and FOMs for two-dimensional arrays of spherical Si-SiO_2_ nanoparticles.

period of the structure	nanoparticle radius	resonance wavelength	FOM
301.7 nm	45 nm	445 nm	1/66
360 nm	50 nm	526 nm	2/5
430 nm	70 nm	636 nm	2/44

**Figure 2:**
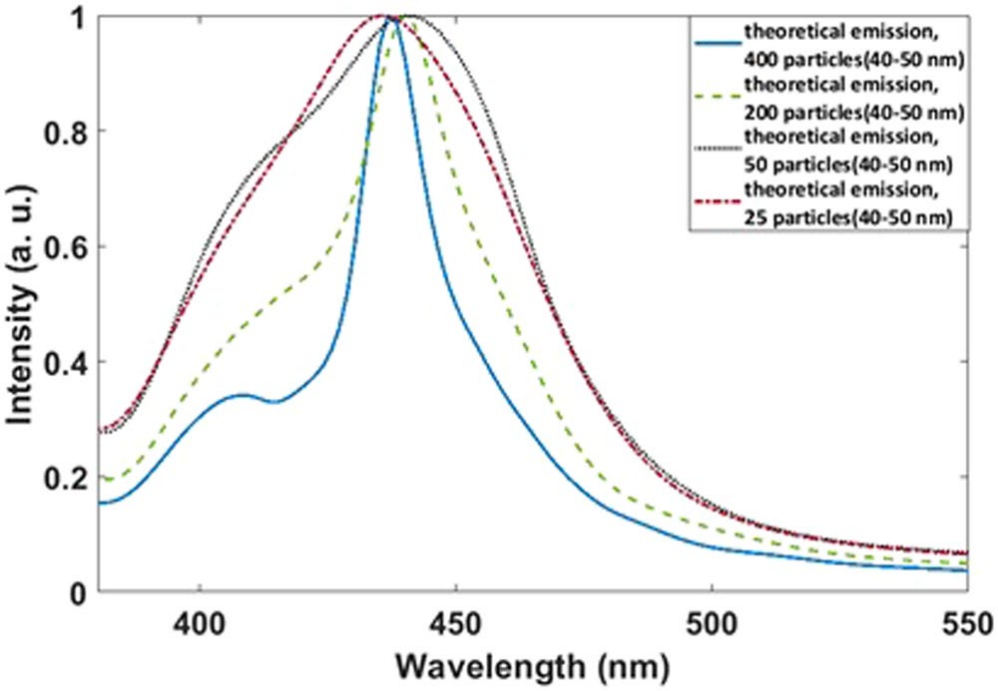
Calculated emission spectra for 400, 200, 50 and 25 nm.

To validate if our solution processed material approaches an ideal array system we simulated nanoparticles in perfect arrays, in quasi-array system and randomly oriented systems thus allowing comparison of theoretical and experimental results. In a first approach, we simulated an array of 400 nanoparticles with random sizes between 40–50 nm and relatively uniform dispersion. As the calculated emission spectra in Fig. 2 show, there are two absorption peaks at 400 nm and 450 nm. With decreasing the number of nanoparticles to 200, the intensity of the peak at 400 nm increases and it overlaps with the peak at 450 nm. With decreasing the number of particles to 50, increasing in the intensity of the peak at 400 nm is more and the overlapping of it with the peak at 450 nm makes a broad peak. For a system with 25 particles, the peak at 400 nm disappears and we can see only one broad peak with the maximum at 450 nm.

**Figure 3:**
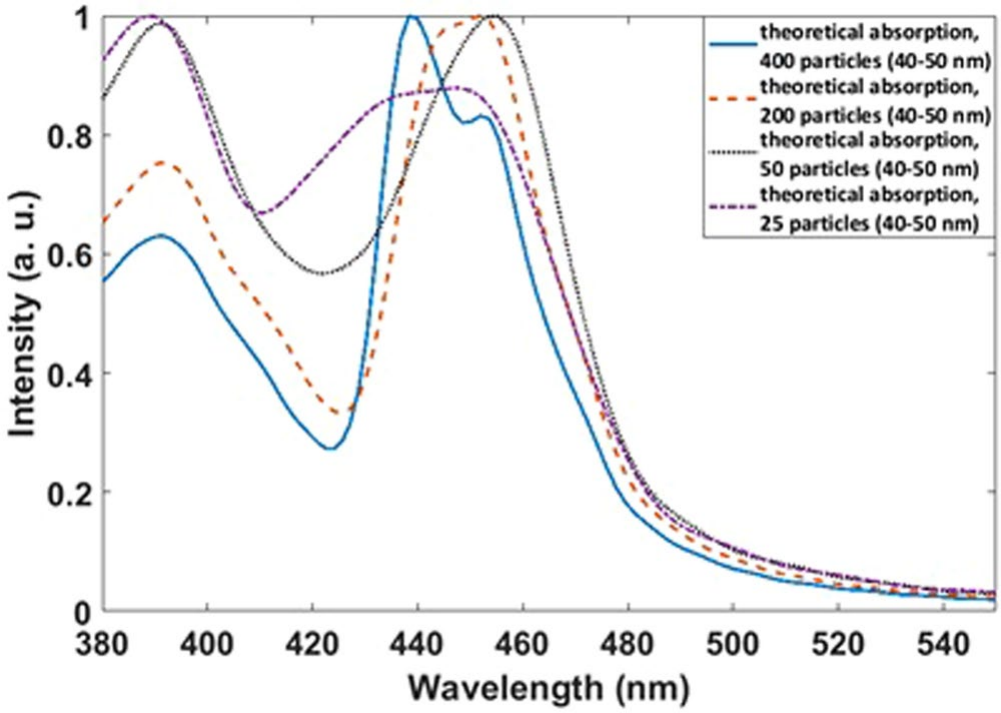
Calculated absorption spectra for 400, 200, 50 and 25 nm.

In the corresponding calculated absorption spectra in Fig. 3 broadening of the peaks at 400 nm is nearly the same. However, in the case of the peak at 450 nm, for 400 particles, the peak has a shoulder at 460 nm. For 25 particles the peak is broad as observed for the emission.

**Figure 4:**
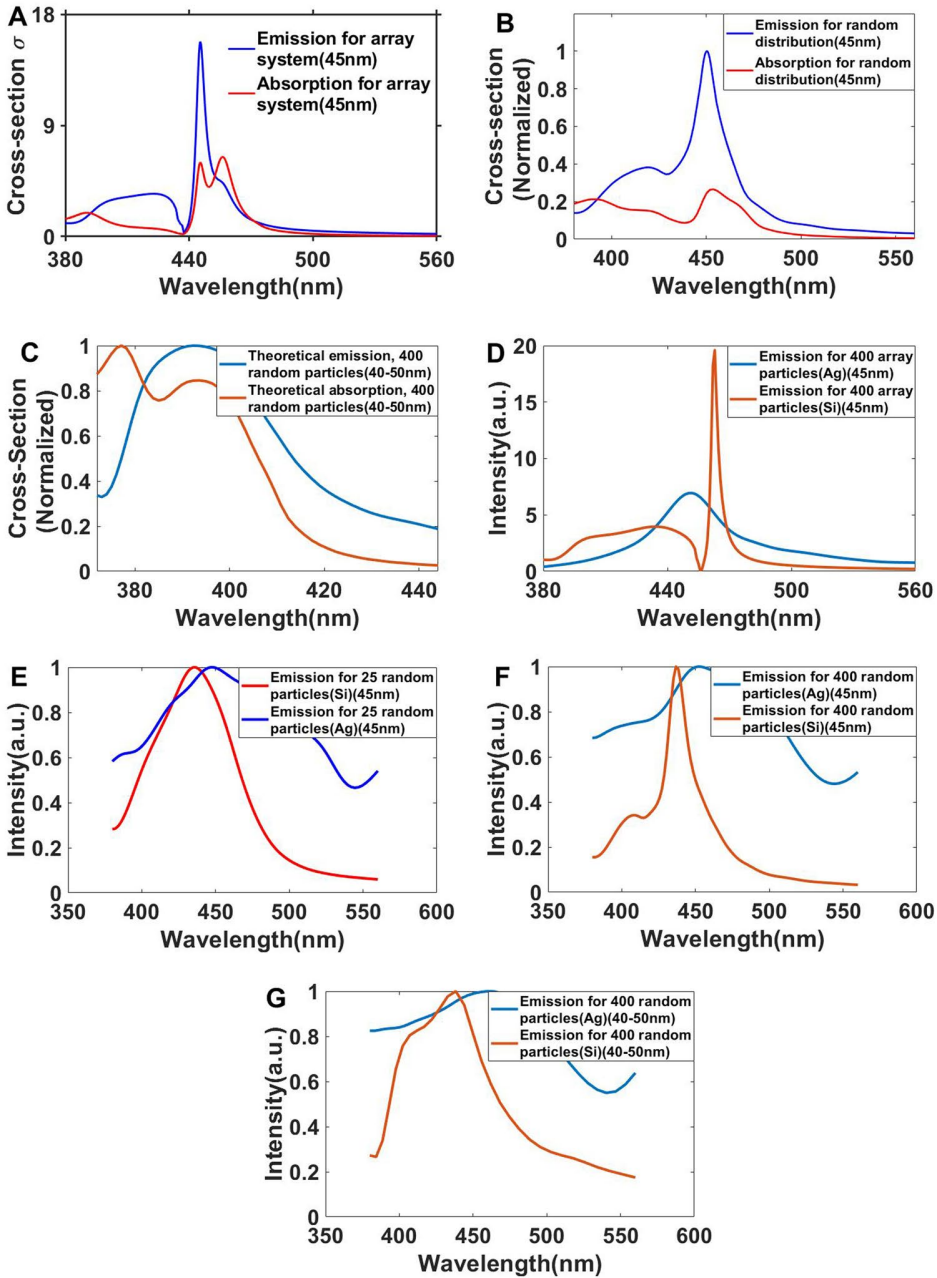
Calculated absorption and emission cross-sections for (**A**) a perfect array of Si-SiO_2_ particles, (**B**) randomly distributed uniform Si-SiO_2_ particles (45 nm), (**C**) a random distribution with different particle sizes and comparison between emissions of Si-SiO_2_ and Ag nanoparticles in (**D**) a perfect array, (**E**) for 25 particles in a quasi-periodic array, (**F**) for a quasi-periodic array of 400 particles, and (**G**) for a random distribution.

Assuming a perfectly random distribution of particles with the size of 45 nm, the cross sections of emission and absorption are different as shown in Fig. 4A. Also, the emission cross-section is very narrower than the random distribution. Choosing the size of particles between 40–50 nm and considering perfect random distribution the simulation yield very broad emissions and absorptions (Fig. 4C).

For comparison between the performances of Si-SiO_2_ nanoparticles as transparent monitor and Ag nanoparticles (reported by Hsu *et al*.^14^) emission spectra for Ag nanoparticles in different distributions as a perfect array, as quasi-periodic and as randomly distributed form are calculated too. Brightness and resolution can be defined by the width and intensity of the emission spectra: narrow peaks indicate high resolution and higher intensity indicates higher brightness. As Fig. 4 shows, the Si-SiO_2_ nanoparticles exhibit very sharp emissions compared with Ag nanoparticles in a perfect array, in quasi-array forms, and even in random distribution. Accordingly, the brightness is higher in the display made from Si-SiO_2_ nanoparticles. At the same time, the Si/SiO_2_ nanoparticles very probably show better performance than Ag nanoparticles in a transparent display. This can be concluded from the highly dispersive permittivity (Palik data) of the Si-SiO_2_ nanoparticles compared with the Ag particles (uniform permittivity in visible band). Interference between scattered light is thus very destructive for Si-SiO_2_ and instructive in the Ag case. Thus, for Si-SiO_2_, we can expect higher intensity and narrow bands.

**Figure 5:**
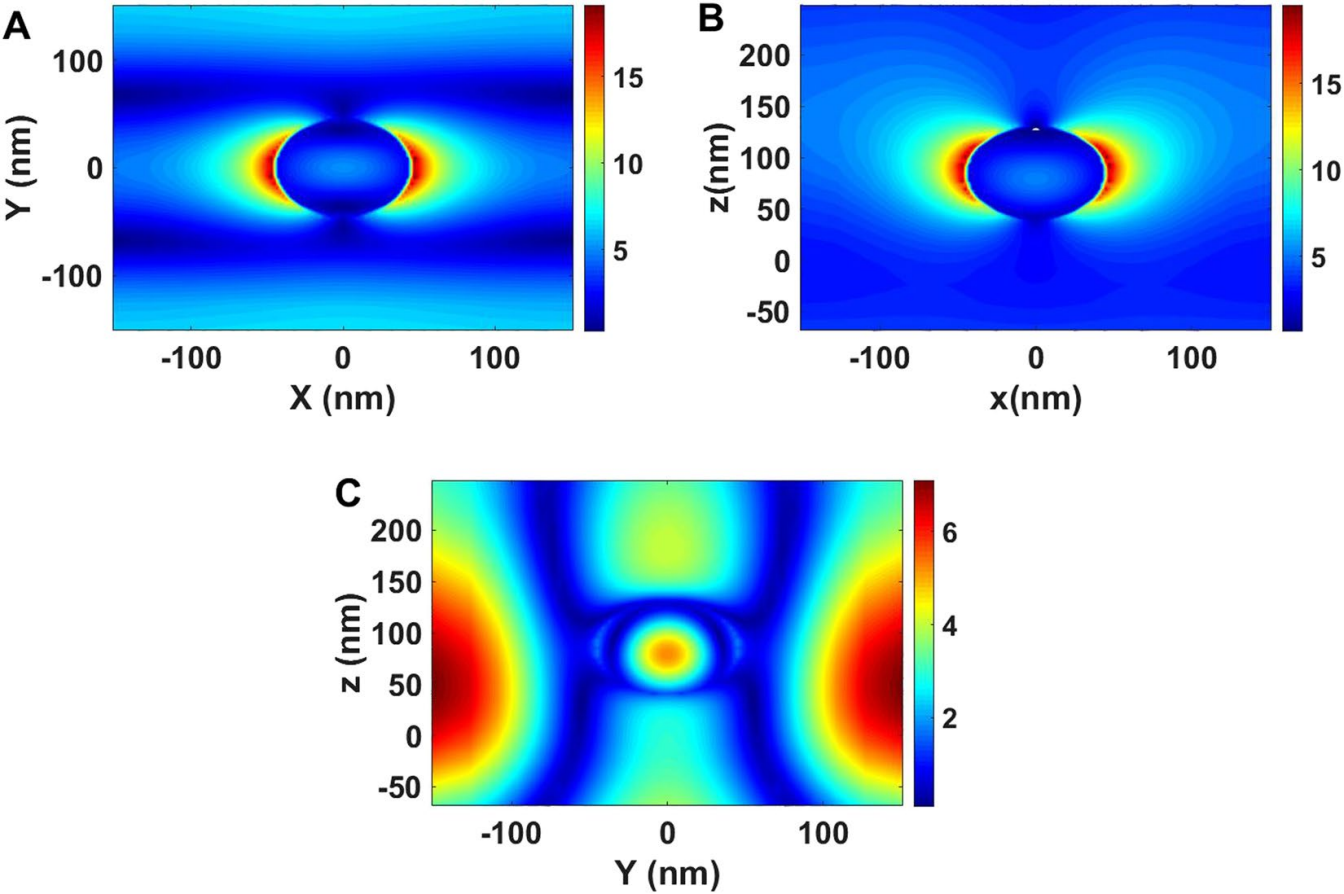
Calculated electric field enhancement profile through the center of a pixel of the Si-SiO_2_ nanoparticle arrays with 45 nm radius and 301.7 nm period in xy plane under plane wave illumination with the wave vector perpendicular to the plane of the array and the polarization in the plane. (**A**) in xy plane (**B**) in the xz planes, (**C**) in the yz planes.

The electric field enhancement profiles through the center of a pixel of the Si-SiO_2_ nanoparticle arrays of 45 nm radius and 301.7 nm period were calculated (Fig. 5). Applying resonance wavelengths, in all cases field enhancement is observed. The electric field in the xy and xz plane is enhanced by more than 15 times (Fig. 5A,B) while in the yz planes the enhancement is about 5 times.

**Figure 6:**
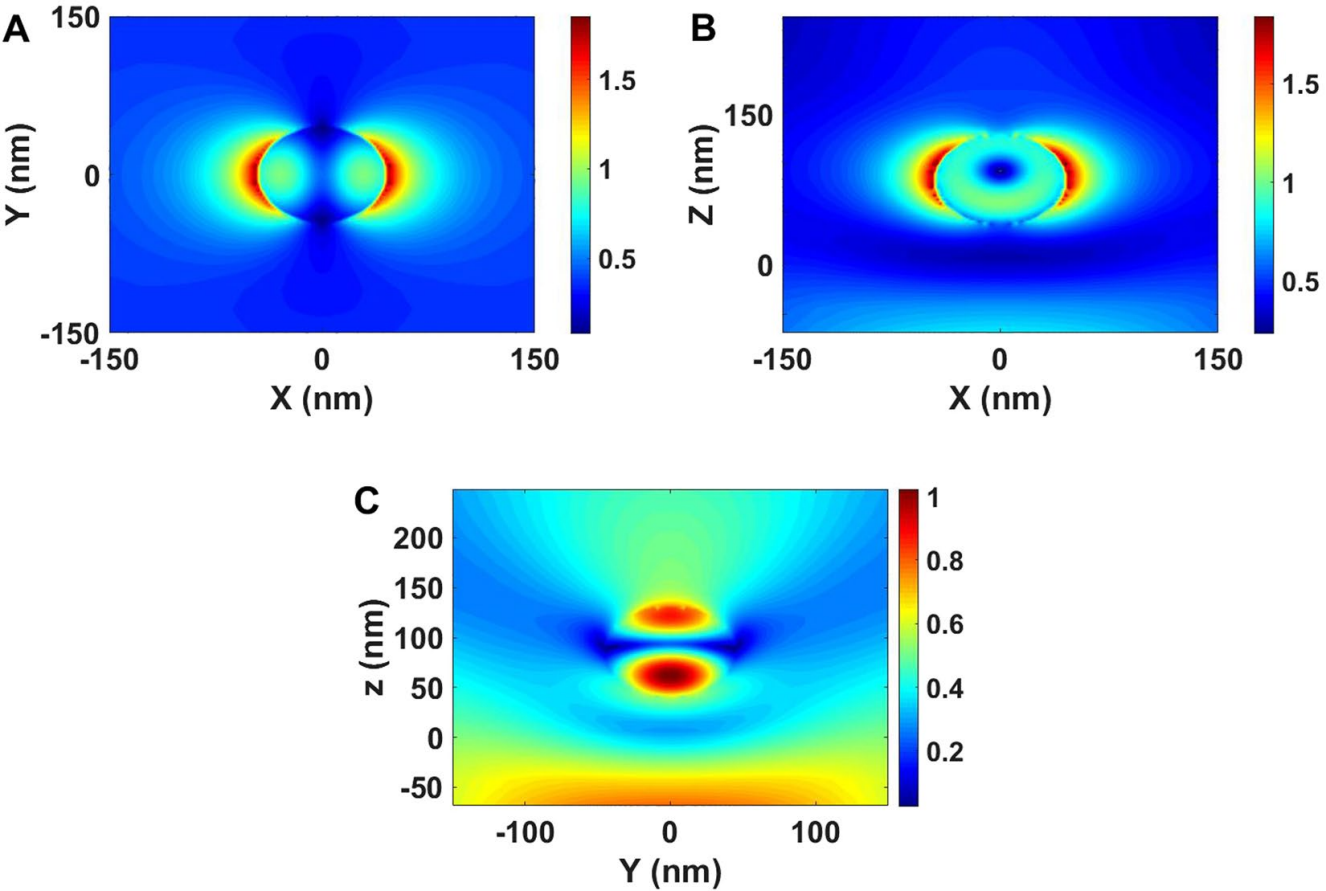
Calculated scattering electric field distribution for Si-SiO_2_ nanoparticles in random distribution situation.

Electric field enhancement profiles through the center of a particle in a random distribution of nanoparticles of 45 nm radius in the XY plane under plane wave illumination with wave vector perpendicular to the plane of the nanoparticles and the polarization in the plane were also calculated. On resonance wavelength, field enhancements in the XY and XZ planes are larger than 1.8 times (Figs 6A and 6B) while the electric field enhancement in the YZ plane is equal with the incident field (Fig. 6C).

**Figure 7:**
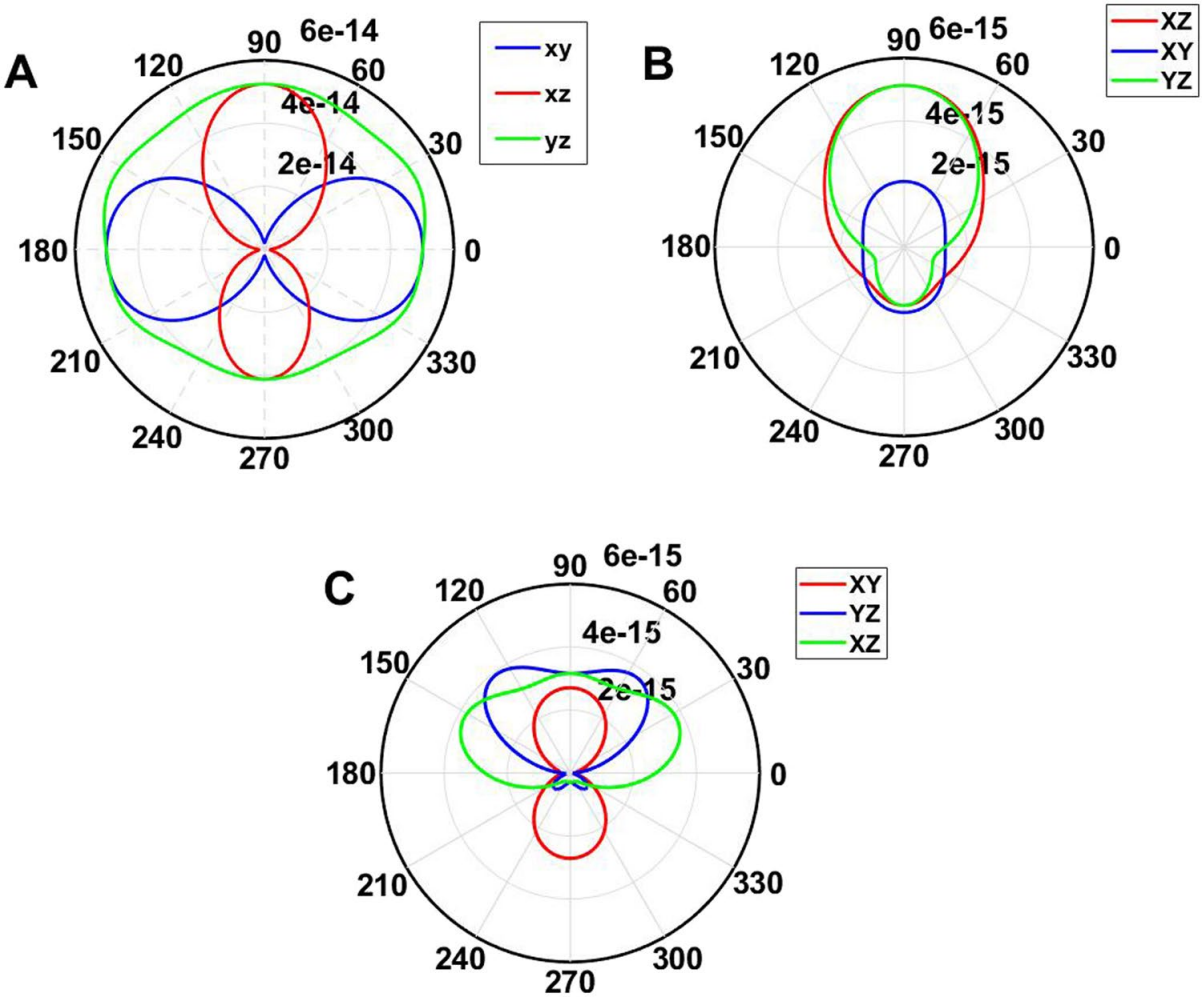
(**A**) Far-field angular scattering behavior of the scattered field of incident light at 445 nm with wave vector perpendicular to the plane of the array (XY) and the polarization in the plane in the XY, XZ and YZ plane for Si/SiO_2_ system. (**B**) Far-field angular scattering behavior of the scattered field of incident light for a single particle in random distribution at 445 nm with wave vector perpendicular to plane (XY) and the polarization in the plane in the XY, XZ and YZ planes. (**C**) Far-field angular scattering behavior of the scattered field of incident light at 445 nm with wave vector perpendicular to the plane of the array (XY) and the polarization in the plane in the XY, XZ and YZ plane for the system based on Ag nanoparticles.

Figure 7 shows the angular distribution of the electric field at resonance wavelength for the array and random distribution structures. The array distribution confirms that the scattered light can be viewed from a wide angle and is better than a random distribution. Figure 7A shows that the diffraction of light in the proposed structure is uniformly distributed in 360 degrees. The viewing angle of the Si/SiO_2_ system in comparison to the silver nanoparticles based monitor (Fig. 7C) reported by Hsu *et al*. is wider.

### Experimental investigations

**Figure 8:**
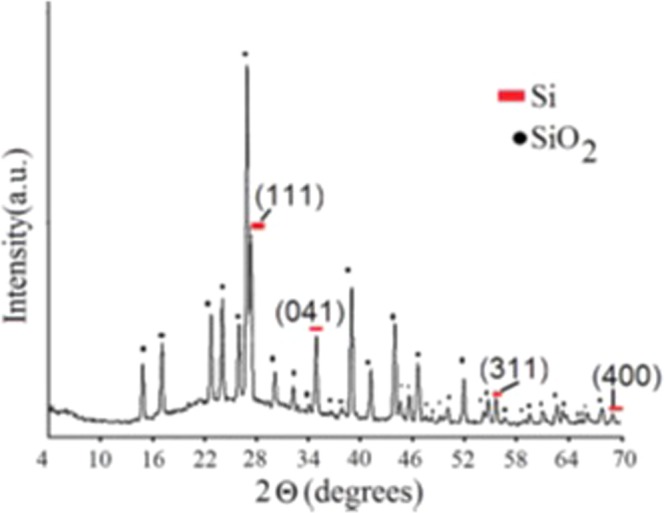
The PXRD pattern of the synthesized Si-SiO_2_ core-shell nanoparticles.

Figure 8 represents the indexed powder XRD pattern of the synthesized Si-SiO_2_ core-shell nanocrystals. The signals at 2ϴ = 28, 47.305, 56.102, and 69.171 can be attributed to silicon in agreement with the JCPDS Card (7440-21-3) for silicon (cubic *F*d-3m, *a* = 5.430 Å). All other signals can be related to SiO_2_ (hexagonal, *P*3221, *a* = *b* = 4.91344 Å and *c* = 5.40524 Å)^42^.

**Figure 9:**
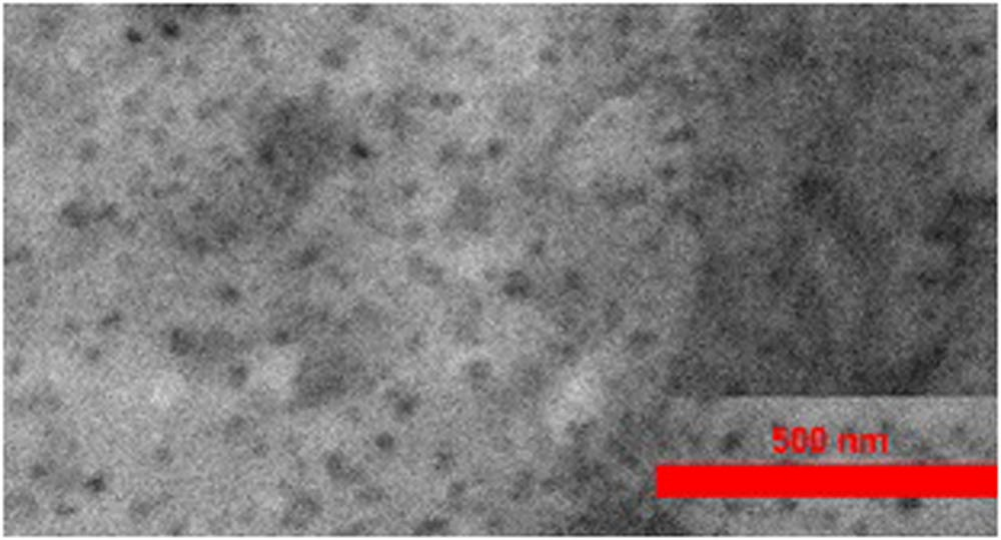
TEM image of the synthesized Si-SiO_2_ nanoparticles.

The TEM image of the synthesized Si-SiO_2_ core-shell nanocrystals (Fig. 9) reveals spherical nanoparticles with sizes about 45 nm.

**Figure 10:**
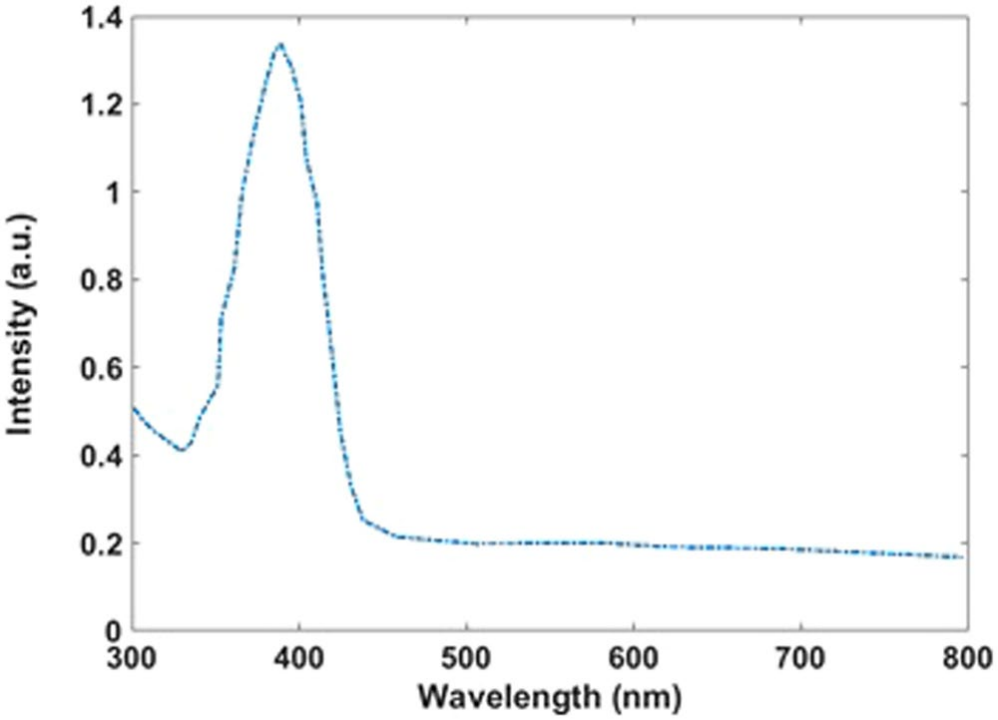
Absorption spectrum of the Si-SiO_2_ nanoparticles.

**Figure 11:**
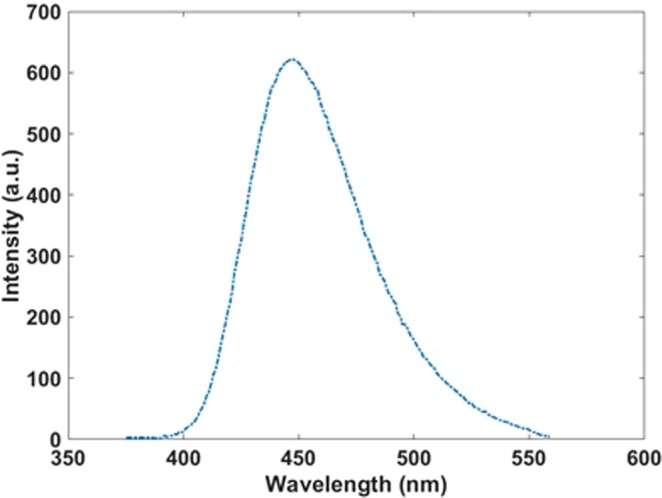
Emission spectrum of the Si-SiO_2_ nanoparticles.

The absorption spectrum of the synthesized Si-SiO_2_ nanoparticles reveals an intense maximum at 400 nm (Fig. 10) and used this wavelength as excitation wavelength to record the photoluminescence (PL) spectrum (Fig. 11) showing an emission maximum around 450 nm in the blue range.

**Figure 12:**
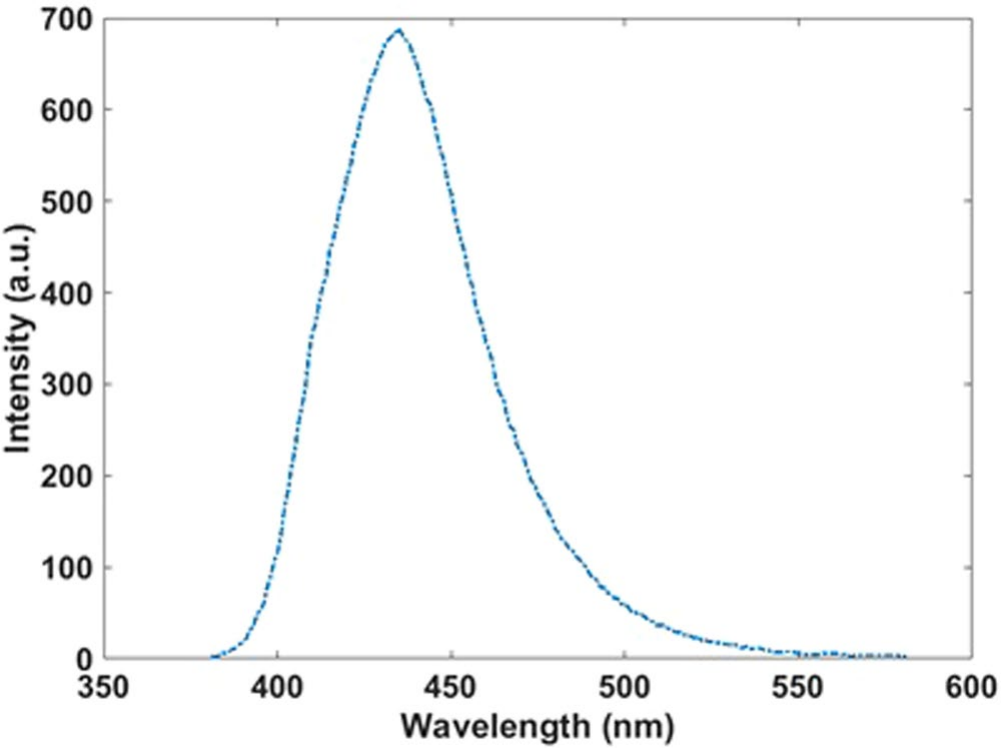
Photoluminescence (PL) spectrum of the Si-SiO_2_ nanoparticles in Polystyrene matrix.

For the fabrication of the transparent monitor, the nanoparticles uniformly merged in polystyrene and then spin-coated on glass. Photoluminescence (PL) bands of these polystyrene-embedded nanoparticles (Fig. 12) are narrower than those of colloids and their structure can be considered as close to a quasi-periodic structure.

**Figure 13:**
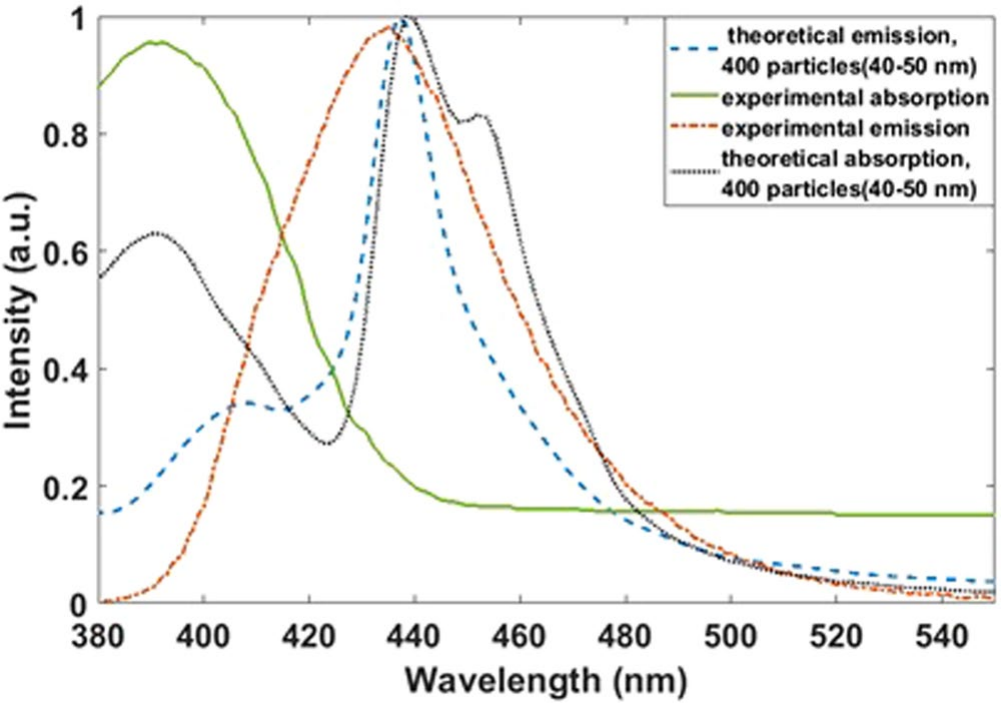
Calculated emission and absorption spectra for 400 particles and experimental spectra.

**Figure 14:**
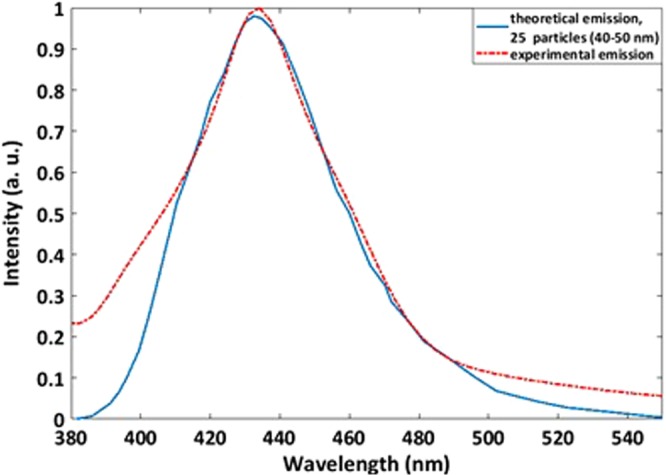
Calculated (blue, 25 particles) and experimental (red) emission spectra.

For comparing the theoretical and experimental results, the obtained absorption and emission spectra for an array with 400 particles are shown in Fig. 13. As can be seen in the absorption spectrum, the peaks at 400 nm and emission peaks at 450 nm in theoretical and experimental results have a good agreement together. However, in the spectrometers, the slit of light is very narrow and narrow beam hits to the sample and it can excite a few particles and the result emission has more agreement with the theoretical absorption result for 25 particles (Fig. 14).

**Figure 15:**
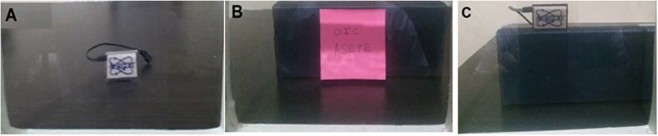
Transparent display for different objects in behind.

After characterization of the nanoparticles and considering the mentioned procedure for making a transparent display, nanoparticles in the colloidal form spin-coated on 18 × 18 cm^2^ glass screen. After drying the polystyrene including nanoparticles on the glass, the following experiments did on it. In the first part, Fig. 15, we examined transparency of the display on white light.

**Figure 16:**
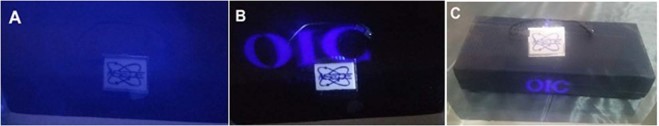
(**A**) Scattering of blue light from the whole transparent screen, (**B**), projecting OIC logo on transparent display and (**C**) projecting OIC in white light ambient.

Images of ASEPE logo on the transparent display under illuminations by blue, Green and Red lights are examined. The results declared that the monitor only is excited by the blue light (Fig. 16). As Fig. 16 shows, the contrast of the image in dark media is high; however, in white light (normal ambient light), the image is distinguishable too. In both Green and Red illuminations actually, there is no image on the screen. For a comparison between white paper as a screen and transparent display, we presented an image on white paper too.

## Conclusions

Theoretical results show that with a periodic structure we can get a sharper emission and absorption. This helps to reach a high-resolution image when we use a structure of transparent monitor. This is because of constructive interference of emitted photons in periodic like structures. However, with solution-processed method reaching the periodic structure is difficult and practically quasi-periodic structure is considered and simulated too. For the evaluation of this results, we used polystyrene medium for dispersing uniform nanoparticles and it is observed that the emission peak appeared in this media is sharper than the spectrum obtained by nanoparticles without polystyrene media. And the broadening of the peak is reached from 38.6 nm to 32.6 nm. This means; we have a band with 6 nm narrower than the emission of quantum dots without polystyrene media for the same quantum dots; it is clear that if the particles are non-uniform the broadening of the peak will be broader.

## Methods

### Synthesis of Si/SiO_2_ nanomaterials

Silicon quantum dots were synthesized in reverse micelles by solution-phase reduction of SiCl_4_ using LiAlH_4_. All experiments were carried out in a nitrogen atmosphere to prevent the oxidation of the silicon. In a typical experiment, while stirring, a solution of 5 mL of SiCl_4_ in 20 mL of 1,2-dimethoxyethane was added dropwise to 30 mL of 1 M solution of LiAlH_4_ in THF. 0.1 ml tetraethyl orthosilicate is added on the solution and stirred for 1 h. Then 1 cc, ammonia 0.001 M added on the solution and stirred for 1 h then anhydrous methanol (20 mL) was slowly added to quench any excess reducing agent. The obtained material transferred to a Teflon lined steel reactor and treated at vacuum conditions for 2 h at 120 °C. The obtained materials were centrifuged and separated for the fabrication of a transparent monitor. For large scale synthesis of nanoparticles with good quality, nanoparticles should be synthesized in diluted conditions and evaporate of the solvent after synthesis to reach desired concentration.

### Fabrication method

Obtained nanomaterial dispersed in toluene and added in the Polystyrene dissolved in toluene. The obtained viscose solution put on a glass and dried for 12 h.

**Figure 17:**
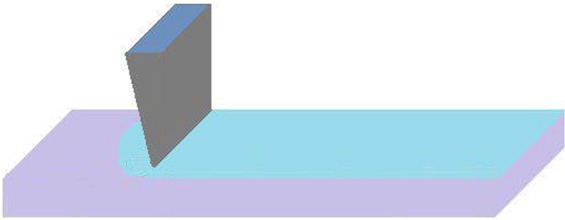
Doctor blade synthesis method.

In the large scale, the doctor blade method is the simplest and widely used method for depositing of the solution including nanoparticles on the substrate. The method is also called slot coating in its programmed version. With this method, the film can be layered in desirable thickness (Fig. 17).

## References

[1] Li, J., Sharlin, E., Greenberg, S. & Rounding, M. In CHI'13 Extended Abstracts on Human Factors in Computing Systems. *ACM conference* 1665–1670 (2013).

[2] Wu, W., Blaicher, F., Yang, J., Seder, T. & Cui, D. Designing the Car iWindow: Exploring Interaction through Vehicle Side Windows. *ACM conference* 21–28 (2009).

[3] Li J, Greenberg S, Sharlin E (2017). Two-sided transparent display as a collaborative medium. University of Calgary. Int. J. Man.-Mach. Stud..

[4] Görrn P (2006). Towards see‐through displays: fully transparent thin‐film transistors driving transparent organic light‐emitting diodes. Adv. Mater..

[5] Ju S (2007). Transparent active matrix organic light-emitting diode displays driven by nanowire transistor circuitry. Nano Lett..

[6] Park SHK (2009). Transparent and photo‐stable ZnO thin‐film transistors to drive an active matrix organic‐light‐emitting‐diode display panel. Adv. Mater..

[7] Newman, R. L. Head-Up Displays: Designing the Way Ahead. ISBN-13: 978-0291398116, *Routledge, London* (1995).

[8] Goldenberg JF, McKechnie TS (1985). Diffraction analysis of bulk diffusers for projection-screen applications. J. Opt. Soc. Am. A..

[9] Kawakami T, Katagiri B, Ishinabe T, Uchida T (2012). High-Resolution Multi-View Projection Display With a Quantized-Diffusion-Angle Screen. J. Disp. Technol..

[10] Lopez OA, McKittrick J, Shea LE (1997). Fluorescence properties of polycrystalline Tm^3+^-activated Y_3_Al_5_O_12_ and Tm^3+^-Li^+^ co-activated Y_3_Al_5_O_12_ in the visible and near IR ranges. J. Lumin..

[11] Yi GS, Chow GM (2007). Water-Soluble NaYF_4_:Yb,Er(Tm)/NaYF_4_/Polymer Core/Shell/Shell Nanoparticles with Significant Enhancement of Upconversion Fluorescence. Chem. Mater..

[12] Liu, Z. *et al*. Monodisperse silica nanoparticles encapsulating upconversion fluorescent and superparamagnetic nanocrystals. *Chem. Commun*. 694–696 (2008). 10.1039/b715402j 18478693

[13] Downing E, Hesselink L, Ralston J, Macfarlane RA (1996). Three-color, solid-state, three-dimensional display. Science.

[14] Hsu CW (2014). Transparent displays enabled by resonant nanoparticle scattering. Nat. Commun..

[15] Sun S (2017). All-Dielectric Full-Color Printing with TiO_2_ Metasurfaces. ACS Nano.

[16] Haynes CL, McFarland AD, Zhao LL, Duyne RPV, Schatz GC (2003). Nanoparticle optics: the importance of radiative dipole coupling in two-dimensional nanoparticle arrays. J. Phys. Chem. B.

[17] Bohren, C. F. & Huffman, D. R. Absorption and Scattering of Light by Small Particles. Absorption and Scattering of Light by Small Particles. *ISBN 0-471-29340-7. Wiley-VCH*, pp 544 (1998).

[18] Newton, R. G. Scattering theory of waves and particles. *Springer Science & Business Media* (2013).

[19] Jensen T, Kelly L, Lazarides A, Schatz GC (1999). Electrodynamics of noble metal nanoparticles and nanoparticle clusters. J. Clust. Sci..

[20] Draine BT, Flatau PJ (1994). Discrete-dipole approximation for scattering calculations. J. Opt. Soc. Am. A..

[21] Novotny L, Pohl D, Hecht B (1995). Scanning near-field optical probe with ultrasmall spot size. Opt. Lett..

[22] Taflove, A. & Hagness, S. C. Computational electrodynamics: the finite-difference-time-domain method. *Artech House* (2005).

[23] Barber, P. W. & Hill, S. C. Light scattering by particles: computational methods. Vol 2, *World scientific* (1990).

[24] Mackowski DW, Mishchenko MI (1996). Calculation of the T matrix and the scattering matrix for ensembles of spheres. J. Opt. Soc. Am. A..

[25] Masadeh AS (2016). Total scattering atomic pair distribution function: new methodology for nanostructure determination. J. Exp. Nanosci..

[26] Khare HS, Burris DL (2010). A quantitative method for measuring nanocomposite dispersion. Polymer.

[27] Tribelsky MI, Lukyanchuk BS (2006). Anomalous light scattering by small particles. Phys. Rev. Lett..

[28] Ruan Z, Fan S (2010). Superscattering of light from subwavelength nanostructures. Phys. Rev. Lett..

[29] Hamam RE, Karalis A, Joannopoulos J, Soljačić M (2007). Coupled-mode theory for the general free-space resonant scattering of waves. Phys. Rev. A..

[30] Ruan Z, Fan S (2011). Design of subwavelength superscattering nanospheres. Appl. Phys. Lett..

[31] Li S, Lin MM, Toprak MS, Kim DK, Muhammed M (2010). Nanocomposites of polymer and inorganic nanoparticles for optical and magnetic applications. Nano Rev..

[32] Zhao L, Kelly KL, Schatz GC (2003). The extinction spectra of silver nanoparticle arrays: influence of array structure on plasmon resonance wavelength and width. J. Phys. Chem. B.

[33] Zou S, Schatz GC (2004). Narrow plasmonic/photonic extinction and scattering line shapes for one and two dimensional silver nanoparticle arrays. J. Chem. Phys..

[34] Malynych S, Chumanov G (2003). Light-induced coherent interactions between silver nanoparticles in two-dimensional arrays. J.Am. Chem. Soc..

[35] Haes AJ, Zou S, Schatz GC, Van Duyne RP (2004). A nanoscale optical biosensor: the long range distance dependence of the localized surface plasmon resonance of noble metal nanoparticles. J. Phys. Chem. B.

[36] Zou S, Janel N, Schatz GC (2004). Silver nanoparticle array structures that produce remarkably narrow plasmon lineshapes. J. Chem. Phys..

[37] Palik, E. D. Handbook of Optical Constants of Solids, Author and Subject Indices for Volumes I, II, and III. Elsevier (1998).

[38] Royer P, Goudonnet J, Warmack R, Ferrell T (1987). Substrate effects on surface-plasmon spectra in metal-island films. Phys. Rev. B.

[39] Foss CA, Hornyak GL, Stockert JA, Martin CR (1994). Template-synthesized nanoscopic gold particles: optical spectra and the effects of particle size and shape. J.Phys. Chem..

[40] Tomchuk P, Tomchuk B (1997). Optical absorption by small metallic particles. J. Exp. Theor. Phys..

[41] Ghoshal A, Kik PG (2009). Excitation of propagating surface plasmons by a periodic nanoparticle array: Trade-off between particle-induced near-field excitation and damping. Appl. Phys. Lett..

[42] Sayyedfattahi SJ (2014). Novel, and Simple Solution-processed MIS Ultraviolet (UV) Detector Based on Core-Shell Si/SiO_2_ Nanocrystals. J. Electron. Mater..

